# Isolation of *Clostridium difficile* and molecular detection of binary and A/B toxins in faeces of dogs

**Published:** 2016

**Authors:** M. Ghavidel, H. Salari Sedigh, J. Razmyar

**Affiliations:** 1 Ph.D. Student in Bacteriology, Department of Microbiology, Faculty of Medicine, Mashhad University of Medical Sciences, Mashhad, Iran;; 2 Department of Clinical Sciences, Faculty of Veterinary Medicine, Ferdowsi University of Mashhad, Mashhad, Iran

**Keywords:** *Clostridium difficile*, Dog, Molecular detection

## Abstract

The aim of this study was to isolate *Clostridium difficile* from dogs’ faeces, and to study the frequency of its virulence genes. A total of 151 samples of dogs’ faeces were collected. The isolation of *C. difficile* was performed by using the bacterial culture methods followed by DNA extraction using boiling method. Multiplex PCR method was performed for identification of *tcdA*, *tcdB*, *cdtA* and *cdtB* genes and single method was carried out for detection of *tcdC*. Twelve samples (7.9%) were positive in bacteriological assay and based on molecular assay, 66.7% of the isolates (8 of 12 *C. difficile* isolated) had shown tcdA^+^, tcdB^+^ profile. This is the first investigation on molecular assay of *C. difficile* in Iran’s dog population.

## Introduction

*Clostridium difficile* is a Gram-positive spore-forming anaerobic bacillus which has been identified as a main bacterial pathogen in both human and animals’ intestine. It is a common cause of enteritis in a variety of animal species (Doosti and Mokhtari-Farsani, 2014 ▶). In addition, some reports have recently raised the importance of wild animals as a reservoir of *C. difficile* for humans and domestic animals.

A number of bacterial organisms commonly associated with diarrhea in dogs and cats include *Salmonella*, *Campylobacter*, *Clostridium perfringens* and *C. difficile* (Marks et al., 2011 ▶).

Two large clostridial toxins A and B (TcdA and TcdB) were among the main virulence factors. TcdA and TcdB are strong cytotoxic enzymes damaging the human colonic mucosa (Deneve et al., 2009 ▶).

TcdA and TcdB in the pathogenicity locus are controlled by two regulators, TcdR and TcdC. TcdR is an alternative sigma factor which positively regulates transcription of *tcdA* and *tcdB* while TcdC may function as an anti-sigma factor impeding the activity of TcdR, although some researches have reported that TcdC does not influence toxin production (McKee et al., 2013 ▶).

The *C. difficile* ADP-ribosyltransferase was a binary toxin consisting of two independently coded protein components: a binding component (CDTb) and an enzymatic component (CDTa) which catalyzes the ADP-ribosylation of monomeric action, inducing alterations in the cytoskeleton (Marks and Kather, 2003 ▶).

In dogs, pathogenicity and the importance of *C. difficile* is not fully understood as yet. Clinical signs that have been associated with canine *C. difficile* infection range from asymptomatic carrier to a potentially fatal acute hemorrhagic diarrheal syndrome (Marks et al., 2011 ▶).

A simple and quick method is required in order to distinguish toxigenic and non-toxigenic strains of *C. difficile* in dogs. The objective of the current study was to investigate the molecular characteristics of various isolates of *C. difficile* isolated from diarrheic and non-diarrheic dogs, through the use of toxin gene profiling.

## Materials and Methods

A total of 151 faecal samples was collected from 151 dogs, 131 of which were apparently healthy and 20 were diarrheic. The samples from diarrheic dogs were obtained directly from the rectum, in a veterinary teaching clinic at the time of consultation and were only collected from dogs in which the main motivation for the consultation was the occurrence of diarrhea. There were 60 male and 91 female dogs, aged from 3 months to 11 years.

Isolation and identification of *C. difficile* were performed according to standard procedures (Fedorko et al., 1997 ▶; Pituch et al., 2002 ▶). Two or three passages were done to obtain the pure culture. Reference strains of *C. difficile* were used as positive controls.

A single colony of each strain was suspended in 100 µL distilled water, boiled for 10 min and then centrifuged at 10,000 × g for 10 min. The supernatants were collected carefully and used as template DNA for PCR (Aldous et al., 2005 ▶).

A 5-plex PCR was performed for the detection of tcdA, tcdB, cdtA, cdtB and 16S rDNA based on Persson et al. (2008) ▶. The tcdC analysis was performed based on Antikainen et al. (2009) ▶, primer list was shown in Table 1. PCR products were fractionated by electrophoresis in 1.5% agarose gels. Data analysis was performed by using SPSS software (SPSS 19.0). Correlation between positive bacteria samples and diarrhea, gender and age was assessed by Chi-square. A p-value <0.05 was considered significant.

## Results

Twelve *C. difficile* (7.9%) were isolated from dog faeces in Columbia agar. Twelve *C. difficile* which have been selected for multiplex PCR. The isolation rate was as follows: diarrheic dogs (4/20: 20%) and healthy dogs (8/131: 6.1%). Out of 91 female dogs 10 (11.8%), and of 60 male dogs 2 (3.2%), were positive in culture. Ten isolates belonged to dogs aged below 3 years old (8.3%) and 2 isolates in dogs above 3 years old (6.6%).

All 12 isolates were confirmed to have 1062 bp band (16S rDNA) of *C. difficle* (7.9%) (Table 2). *Clostridium difficile* toxins A and B were identified in faeces of 8 (66.7%) samples of 12 isolates and four isolates (33.3%) were non-toxigenic (*tcdA*^-^,* tcdB*^-^).

One isolate possessed binary toxin (*cdtA*, *ctdB*) in addition to *tcdA* and *tcdB* belonging to non-diarrheic dog. *TcdC* band includes: (139, 121, 100 or 85 bp) 2 isolates with 85 bp band and 2 *tcdC* gene negative isolates which were also 16s rDNA positive and non-toxigenic. Eight isolates have 139 bp bands, 4 of which were related to diarrheic dogs.

There was not any significant correlation between positive *C. difficle* and dogs’ diarrhea (P=0.055) nor between positive *C. difficle* and gender (P=0.085). In this study, dogs were thus divided into three age categories:

Under 1 year, 1-3 years** and **More than 3 years in which correlations between age and positive *C. difficle* isolated were not significant (P=0.856).

**Table 1:** Multiplex PCR primer and single PCR primer in this study

Analysis	Gene target	Primer name	Sequence (5´-3´)	Primer concentration (µM)	Amplicon size (bp)	Reference
**5-plex PCR**	* tcdA*	tcdA-F3345	GCATGATAAGGCAACTTCAGTGGTAa	0.6	629	Persson *et al*. (2008)
		tcdA-R3969	AGTTCCTCCTGCTCCATCAAATG	0.6		
	* tcdB*	tcdB-F5670	CCAAARTGGAGTGTTACAAACAGGTG	0.4	410	Persson *et al*. (2008)
		tcdB-R6079A	GCATTTCTCCATTCTCAGCAAAGTA	0.2		
	* cdtA*	cdtA-F739A	GGGAAGCACTATATTAAAGCAGAAGC	0.05	221	Persson *et al*. (2008)
		cdtA-F739B	GGGAAACATTATATTAAAGCAGAAGC	0.05		
		cdtA-R958	CTGGGTTAGGATTATTTACTGGACCA	0.1		
	* ctdB*	ctdB-F617	TTGACCCAAAGTTGATGTCTGATTG	0.1	262	Persson *et al*. (2008)
		cdtB-R878	CGGATCTCTTGCTTCAGTCTTTATAG	0.1		
	16S rDNA	PS13	GGAGGCAGCAGTGGGGAATA	0.05	1062	Persson *et al*. (2008)
		PS14	TGACGGGCGGTGTGTACAAG	0.05		
**tcdC analysis**	* tcdC*	tcdC-121-F	AAGCTATTGAAGCTGAAAATC	0.15	139 (intact)	Antikainen *et al*. (2009)
		tcdC-121-R	GCTAATTGGTCATAAGTAATACC	0.15		

**Table 2:** Identification of *C. difficile* isolated form canine faecal samples complemented with other data

Dogs	*Closteridium difficile*				Total
	16s RND tcdA^+^, tcdB^+^, tcdC^+^ (139 bp)	16s RND tcdA^+^, tcdB^+^, tcdC^+ ^(139 bp), ctdAB^+^	16s RND tcdA^+^, tcdB^+^, tcdC^+^ (85 bp)	16s RND tcdA^-^, tcdB^-^, tcdC^-^	
Diarrheic	4/4 (100%)	-	-	-	4/4 (100%)
	4/20 (20%)	-	-	-	4/20 (20%)
Non-diarrheic	3/8 (37.5%)	1/8 (12.5%)	2/8 (25%)	2/8 (25%)	8/8 (100%)
	3/131 (2.2%)	1/131 (0.8%)	2/131 (1.5%)	2/131 (1.5%)	8/131 (6.1%)
Total	7/12 (58.3%)	1/12 (8.3%)	2/12 (16.7%)	2/12 (16.7%)	12/151 (7.9%)
	7/151 (4.6%)	1/151 (0.7%)	2/151 (1.3%)	2/151 (1.3%)	
Female	6/10 (60%)	-	2/10 (20%)	2/10 (20%)	10/10 (100%)
	6/91 (6.6%)	-	2/91 (2.2%)	2/91 (2.2%)	10/91 (11%)
Male	1/2 (50%)	1/2 (50%)	-	-	2/2 (100%)
	1/60 (1.7%)	1/60 (1.7%)	-	-	2/60 (3.3%)
Total	7/12 (58.3%)	1/12 (8.3%)	2/12 (16.7%)	2/12 (16.7%)	12/12 (100%)
	7/151 (4.6%)	1/151 (0.7%)	2/151 (1.3%)	2/151 (1.3%)	12/151 (7.9%)
Age ≤3 years	7/10 (70%)	-	1/10 (10%)	2/10 (20%)	10/10 (100%)
	7/121 (5.8%)	-	1/121 (0.8%)	2/121 (1.6%)	10/121 (8.2%)
Age >3 years	-	1/2 (50%)	1/2 (50%)	-	2/2 (100%)
	-	1/30 (3.3%)	1/30 (3.3%)	-	2/30 (6.7%)
Total	7/12 (58.3%)	1/12 (8.3%)	2/12 (16.7%)	2/12 (16.7%)	12/12 (100%)
	7/151 (4.6%)	1/151 (0.7%)	2/151 (1.3%)	2/151 (1.3%)	12/151 (7.9%)

## Discussion

*Clostridium* *difficile* has been isolated from almost all mammals (Dabard et al., 1979 ▶; Frazier et al., 1993 ▶; Hasanzade et al., 2013 ▶).

In Iran several studies have been performed for detection of *C. difficile* (Jalali et al., 2012 ▶; Fooladi et al., 2014 ▶; Rahimi et al., 2014 ▶) but this study is the first investigation on *C. difficile* isolated from dog population in Iran. Many researches on animals concentrate on the presence of the bacterium in healthy animals. Investigation on the role of household pets as a possible reservoir of *C. difficile* revealed that both healthy and diseased dogs and cats can shed spores of *C. difficile* (Riley et al., 1991 ▶).

Colonization of humans by *C. difficile* can produce enteric symptoms termed CDI, ranging from asympto-matic intestinal colonization to diarrhea. The most common transmission routes of *C. difficile* include: direct transmission from human to human, direct contact with the animals and environment, aerosol transmission and consuming contaminated food and water (Ghose, 2013 ▶).

There have been several studies worldwide aimed at isolating and molecular typing *C. difficile* in dogs (Marks et al., 2002 ▶; Kevin et al., 2007 ▶; Clooten et al., 2008 ▶; Koene et al., 2011 ▶; Ossiprandi et al., 2012 ▶; Silva et al., 2013 ▶).

In this study, 12 isolates of *C. difficile* were isolated from 151 dogs (7.9%) in which the number of dogs being *C. difficile* positive was lower than other recent reports (O’Neill et al., 1993 ▶; Marks et al., 2002 ▶; Kevin et al., 2007 ▶), however two other studies have reported a lower rate of *C. difficile* in comparison with the current study (Weese et al., 2001 ▶; Wetterwik et al., 2013 ▶) 2%, 5.7% positive samples, respectively.

In this study, 8 of the 12 isolates (66.6%) were toxigenic (tcdA^+^, tcdB^+^). Our research was similar to that of Ossiprandi et al. (2012 ▶). The last research signifies that 60% of the isolates were toxigenic (tcdA^+^, tcdB^+^). Clooten et al. (2008) ▶ found that 69% of the isolates were toxigenic (tcdA^+^, tcdB^+^).

In the current study, one isolate possessed binary toxin gene which was A^+^ B ^+^ and was derived from non-diarrheic dog. Silva et al. (2013) ▶ found one strain with this characteristic.

In this study, we observed the fact that 4 isolates showed tcdA^+^, tcdB^+^ and tcdC^+^ patterns, while the subjects have shown clinical signs of diarrhea which might be due to the variability of tcdC alleles among toxigenic isolates. There have been reports indicating *C. difficile* isolates being characterized by a non-specific in-frame 18 bp deletion and a specific point deletion at position 117, which results in a frame-shift mutation introducing a stop codon at position 196. This phenomenon leads to a truncated, inactive TcdC protein, the severe truncation of this protein, therefore, seems responsible for the increased toxin production in these pathogenic *C. difficile* isolates which would usually negatively regulate toxin production (Deneve et al., 2009 ▶).
